# *Lactobacillus gasseri* Suppresses the Production of Proinflammatory Cytokines in *Helicobacter pylori*-Infected Macrophages by Inhibiting the Expression of ADAM17

**DOI:** 10.3389/fimmu.2019.02326

**Published:** 2019-10-04

**Authors:** Hanna G. Gebremariam, Khaleda Rahman Qazi, Tanvi Somiah, Sushil Kumar Pathak, Hong Sjölinder, Eva Sverremark Ekström, Ann-Beth Jonsson

**Affiliations:** ^1^Department of Molecular Biosciences, The Wenner-Gren Institute, Stockholm University, Stockholm, Sweden; ^2^Department of Bioscience and Bioinformatics, Khallikote University, Berhampur, India; ^3^Center for Clinical Research Sörmland, Uppsala University, Eskilstuna, Sweden

**Keywords:** *Helicobacter pylori*, infection, inflammation, *Lactobacillus*, ADAM17

## Abstract

The ability of *Helicobacter pylori* to evade the host immune system allows the bacterium to colonize the host for a lifetime. Long-term infection with *H. pylori* causes chronic inflammation, which is the major risk factor for the development of gastric ulcers and gastric cancer. Lactobacilli are part of the human microbiota and have been studied as an adjunct treatment in *H. pylori* eradication therapy. However, the molecular mechanisms by which lactobacilli act against *H. pylori* infection have not been fully characterized. In this study, we investigated the anti-inflammatory effects of *Lactobacillus* strains upon coincubation of host macrophages with *H. pylori*. We found that *Lactobacillus gasseri* Kx110A1 (L. gas), a strain isolated from a human stomach, but not other tested *Lactobacillus* species, blocked the production of the proinflammatory cytokines TNF and IL-6 in *H. pylori*-infected macrophages. Interestingly, L. gas also inhibited the release of these cytokines in LPS or LTA stimulated macrophages, demonstrating a general anti-inflammatory property. The inhibition of these cytokines did not occur through the polarization of macrophages from the M1 (proinflammatory) to M2 (anti-inflammatory) phenotype or through the altered viability of *H. pylori* or host cells. Instead, we show that L. gas suppressed the release of TNF and IL-6 by reducing the expression of ADAM17 (also known as TNF-alpha-converting enzyme, TACE) on host cells. Our findings reveal a novel mechanism by which L. gas prevents the production of the proinflammatory cytokines TNF and IL-6 in host macrophages.

## Introduction

The human-adapted bacterial pathogen, *Helicobacter pylori*, colonizes the stomach of more than half of the world's population. Infection with *H. pylori* is often acquired early in childhood and persists throughout the lifetime of the host, if left untreated. In some cases, long-term carriage of the pathogen increases the risk of developing gastric disorders that include peptic ulcers, gastric adenocarcinoma, and mucosa-associated lymphoid tissue (MALT) lymphoma (1, 2). Upon *H. pylori* infection, the host mounts a vigorous inflammatory response but often fails to eradicate the pathogen leading to persistent infection (3). The majority of *H. pylori*-infected individuals are asymptomatic, but some develop overt diseases. Differences in host genetics are one of the reasons why only certain individuals develop serious infections. For example, polymorphisms that increase the expression of cytokine genes, such as tumor necrosis factor (TNF), IL-1β, and IL-8, have been shown to contribute to the amplification of inflammation (4–6). Moreover, the continuous production of cytokines, chemokines, reactive oxygen species (ROS), and reactive nitrogen species (RNS) from recruited immune cells causes oxidative stress, tissue injury, and DNA damage, which in turn predisposes the host to develop gastric cancer later in life (5, 7, 8).

Macrophages are among the first cell types that *H. pylori* encounters if it invades the gastric epithelial barrier. Macrophages are the primary producers of TNF, and increased levels of TNF have been associated with an increased risk of gastric cancer (9–11). Kaparakis and coworkers showed that short-term depletion of macrophages from mice significantly reduced *H. pylori*-induced gastritis, demonstrating the contributory role macrophages play in the severity of gastric inflammation (11). Depending on environmental stimuli, macrophages polarize to M1 (classically activated macrophages) or M2 (alternatively activated macrophages) phenotypes. M1 macrophages (mostly present during *H. pylori* infection) play an essential role in initiating the host response and eliminating pathogens through the production of proinflammatory mediators and antimicrobial molecules, such as nitric oxide (NO). In contrast, M2 macrophages resolve inflammation and are involved in wound healing and tissue homeostasis (12–14). Although macrophages are efficient at killing *H. pylori*, the pathogen has evolved several strategies to manipulate these effector cells and escape macrophage-mediated killing. For instance, *H. pylori* strains that carry the cag pathogenicity island (Cag-PAI) are able to block phagocytosis (15). *H. pylori* can also survive inside phagosomes when internalized (16, 17). Furthermore, *H. pylori* prevents NO production (18) and induces apoptosis in macrophages (19). The inability of macrophages to clear *H. pylori* produces a vicious cycle of the inflammatory response that eventually leads to peptic ulceration and favors gastric cancer development.

Because antibiotic treatment has become less effective at eradicating *H. pylori* infection, supplementation with probiotics, mainly strains of *Lactobacillus*, has been reported to ameliorate disease and reduce drug side effects (20–23). *Lactobacillus* strains are able to interfere with *H. pylori* virulence mechanisms, either by directly affecting the pathogen through inhibition of its adherence (24, 25), growth (26–28) or expression of virulence genes (24, 29, 30) or through indirectly modulating host cell responses (31, 32). However, the underlying mechanisms by which this occurs are poorly understood. In this study, we investigated whether strains of *Lactobacillus* are able to modulate the inflammatory response induced by *H. pylori* in human macrophages.

Here, we demonstrate a novel anti-inflammatory mechanism of lactobacilli preventing the production of proinflammatory cytokines, TNF, and IL-6, in macrophages. We show that, out of four strains of lactobacilli tested, only L. gas was able to consistently inhibit the production of these cytokines in macrophages. The anti-inflammatory effect of this *Lactobacillus* strain was not *H. pylori*-specific; instead, the lactobacilli acted directly on macrophages by inhibiting the expression of ADAM17 (a disintegrin and metalloprotease 17). ADAM17 (also called TACE, TNF-alpha converting-enzyme) is an essential enzyme that regulates the expression of a vast array of genes through the cleavage of different transmembrane proteins and their receptors to a soluble form (33). The ectodomain regions of TNF, the TNF receptor (TNFR), and the IL-6 receptor (IL-6R), are few examples of substrates that are proteolytically cleaved and released by ADAM17. Contact with host cells was necessary for the *Lactobacillus*-mediated anti-inflammatory function, which suggests that it is not mediated by a secreted product and paves the way for research into the characterization of lactobacilli effector molecules. Overall, the inhibition of proinflammatory cytokine production through ADAM17 may represent a previously unidentified mechanism by which lactobacilli modulate the host immune response.

## Materials and Methods

### Bacterial Strains, Growth Conditions, and Preparation

All *Lactobacillus* strains were isolated from healthy humans. *Lactobacillus gasseri* Kx110A1 (L. gas) and *Lactobacillus* oris Kx112A1 (L. oris), both isolated from gastric biopsies have been described previously (25). *Lactobacillus brevis* ATCC 14869 (L. bre) and *Lactobacillus rhamnosus* GG ATCC 53103 (LGG) were both isolated from feces. Lactobacilli were first grown on Rogosa agar plates and then cultured overnight in MRS broth (Oxoid, Thermo Fisher Scientific) at 37°C with 5% CO_2_. Prior to each experiment, overnight cultures of lactobacilli were washed and resuspended in RPMI 1640 (Thermo Fisher), supplemented with 10% heat-inactivated fetal bovine serum (FBS, Sigma-Aldrich). *H. pylori* strain 67:21, which has been described previously (34), was cultured on Columbia blood agar plates (Acumedia) supplemented with 8% inactivated horse serum and 8% defibrinated horse blood (Håtunalab) at 37°C under microaerophilic conditions. For infection with dead bacteria, heat-killing of lactobacilli was performed by incubating lactobacilli at 95°C for 15 min. Treated samples were then plated on Rogosa agar plates to verify that all bacteria were dead.

### Cell Lines and Culture Conditions

THP-1 (ATCC TIB-202) cells were cultured in RPMI 1640 with 10% FBS at 37°C with 5% CO_2_. To differentiate THP-1 cells into macrophages, cells were cultured in medium supplemented with 0.1 μM phorbol 12-myristate 13-acetate (PMA, Sigma-Aldrich) for 3 days.

### *In vitro* Monocyte Isolation and Polarization

CD14^+^ primary monocytes were isolated as previously described (35) from buffy coats of unidentified healthy donors (Karolinska University Hospital, Stockholm, Sweden). Monocytes were enriched using RosetteSep human monocyte enrichment cocktail (Stem Cell Technologies) and were separated by a Ficoll density gradient (Stem Cell Technologies). For differentiation into macrophages, purified monocytes were cultured in 24-well plates in RPMI 1640 medium containing 10% FBS and recombinant human macrophage colony-stimulating factor (M-CSF, 50 ng/ml, Immunotools) for 6 days at 37°C with 5% CO_2_. Some of these cells were then polarized to M1 macrophages using *Escherichia coli* LPS (50 ng/ml; Sigma-Aldrich) and into M2 macrophages using IL-4 plus IL-13 (both 50 ng/ml, Immunotools) for 24 h.

### Cell Stimulation With Bacterial Strains

Macrophages differentiated from primary monocytes or THP-1 cells were cultured in 24- or 48-well plates, respectively, to 90–100% confluence. Cells were infected at a multiplicity of infection (MOI) of 50 with *H. pylori* alone or with *H. pylori* in combination with different *Lactobacillus* strains at an MOI of 100 for various time points (2, 4, 6, or 8 h). When different concentrations of lactobacilli were used, lactobacilli at MOIs of 10, 50, 100, and 200 were added to the macrophages in combination with *H. pylori*. For pre-stimulation assays, cells were first incubated with lactobacilli for 2 h, followed by washing to remove unbound bacteria prior to addition of *H. pylori*. To avoid direct contact between lactobacilli and the host cells, Millicell 0.4 μm cell culture inserts (Millipore) were used. In some experiments, cells were treated with *E. coli* LPS (1 μg/ml; Sigma-Aldrich) or *Staphylococcus aureus* LTA (1 μg/ml; Invivogen), alone or together with lactobacilli for 8 h. At each time point, cell culture supernatants were collected and stored at −80°C until enzyme-linked immunosorbent assays (ELISAs) were performed.

### Preparation of Cell Lysates

Cell lysates were prepared as previously described (36). Briefly, infected macrophages were detached from tissue culture plates using the non-enzymatic cell dissociation solution (Sigma). Cells were washed five times and incubated for 1 h in cell lysis buffer [50 mM Tris-HCl, 150 mM NaCl, 0,1% SDS, 1% sodium deoxycholate, 1% Triton X-100, and EDTA-free protease inhibitor cocktail (Roche)]. The lysates were then centrifuged, supernatants were collected, and the protein concentration was quantified using a micro bicinchoninic protein assay kit (Thermo Fisher Scientific).

### ELISA

Levels of human TNF and IL-6 in the cell culture supernatants and ADAM17 in cell lysates were quantified using ELISA kits from Biolegend [TNF (CAT-No:430204) and IL-6 (CAT-No:430501)] and R&D systems (CAT-No:DY930), respectively, according to the manufacturer‘s instructions.

### *H. pylori* Viability Assay

A viability assay was performed as previously described (25). Macrophages differentiated from THP-1 cells were incubated with *H. pylori* or coincubated with both *H. pylori* and lactobacilli as indicated above for 8 h. Cell culture supernatants were collected and stored at 37°C with 5% CO_2_ until the cells were lysed with 1% saponin in cell culture medium for 10 min. Cell supernatants and lysates were pooled together, and viable counts were performed by serial dilution and plating on blood agar plates containing 200 μg/ml bacitracin (Sigma-Aldrich). Blood agar plates were incubated at 37°C under microaerophilic conditions, and colony forming units (cfu) were counted after 4–7 days.

### MTT Cell Viability Assay

THP-1 cell-derived macrophages in 96-well plates were infected with only *H. pylori* or infected simultaneously with *Lactobacillus* strains for 8 h. Unbound bacteria were removed by washing the cells three times. Cells were then incubated with cell culture medium containing 100 mg/ml gentamicin for 2 h. After gentamicin treatment, the supernatants were spread on plates to confirm that all extracellular bacteria were dead. The cells were washed again to remove the antibiotics, and the cell viability was evaluated using a Vybrant® MTT Cell Proliferation kit (Thermo Fisher Scientific), following the manufacturer's instructions. The viability of cells was given as the percentage relative to unstimulated cells.

### Quantitative Real-Time PCR (qPCR)

Cells were incubated with *H. pylori* alone or in combination with *Lactobacillus* strains for 8 h. RNA was extracted using the RNeasy Plus Mini kit (Qiagen) following the manufacturer's instructions. The concentration and purity of the RNA was determined with a NanoDrop 8000, and the RNA was reverse transcribed to cDNA using the SuperScript VILO Mastermix (Thermo Fisher Scientific). qPCR was performed using a LightCycler 480 and SYBR Green I Master kit (Roche) using the primers listed in Table 1. Gene expression levels were normalized against the reference gene human ribosomal protein L37A (RPL37A). Data are presented as the fold change relative to unstimulated cells.

**Table 1:** Primers used in this study.

**Gene**	**Primer sequence (5^**′**^-3^**′**^)**	**References**
*TNF*	Forward: CCTGCCCCAATCCCTTTATT	(37)
	Reverse: CCCTAAGCCCCCAATTCTCT	
*IL-6*	Forward: GGCACTGGCAGAAAACAACC	(37)
	Reverse: GCAAGTCTCCTCATTGAATCC	
*ADAM17*	Forward: GAAGTGCCAGGAGGCGATTA	(36)
	Reverse: CGGGCACTCACTGCTATTACC	
*RPL37A*	Forward: ATTGAAATCAGCCAGCACGC	(37)
	Reverse: AGGAACCACAGTGCCAGATCC	

### Flow Cytometry

Stimulated cells were seeded in a 96-well v-shaped staining plate (Sarstedt) at a concentration of 1 × 10^6^ cells/well. A FACS panel was created to characterize the polarized macrophages. Cells were first stained with the LIVE/DEAD Fixable Dead Cell Stain Kit-Aqua (Life Technologies) according to the manufacturer's instructions. Blocking of the cell surface Fc receptor was performed with 10% human serum. Staining of the cell surface markers was performed using the following antibodies from BioLegend: CD80, CD209, CD206, HLA-DR, and CD11b. After surface staining, cells were washed and fixed/permeabilized with the transcription factor buffer set (BioLegend) according to the manufacturer's instructions. Intracellular blocking was performed with 10% human serum. The cells were then stained for intracellular CD68 using an antibody from BD Biosciences. Stained cells were washed, resuspended in FACS wash buffer and acquired using a FACS verse instrument and FACS Suite software (BD Biosciences). Data analysis was performed using Flow Jo software.

### ADAM17 Blocking Assay

THP-1 cell-derived macrophages were stimulated with *H. pylori* alone or simultaneously with lactobacilli strains in the presence or absence of TAPI-1 (Merck Millipore). After incubation, cell supernatants were collected and TNF levels were quantified using ELISA.

### Statistical Analysis

The GraphPad Prism software, version 8 was used to analyze differences between multiple groups with an analysis of variance (ANOVA) followed by Bonferroni posttest. A *p*-value of < 0.05 was considered statistically significant. Error bars represent the standard deviation.

## Results

### Certain Lactobacilli Attenuate *Helicobacter*-Induced Proinflammatory Cytokines

**Figure 1:**
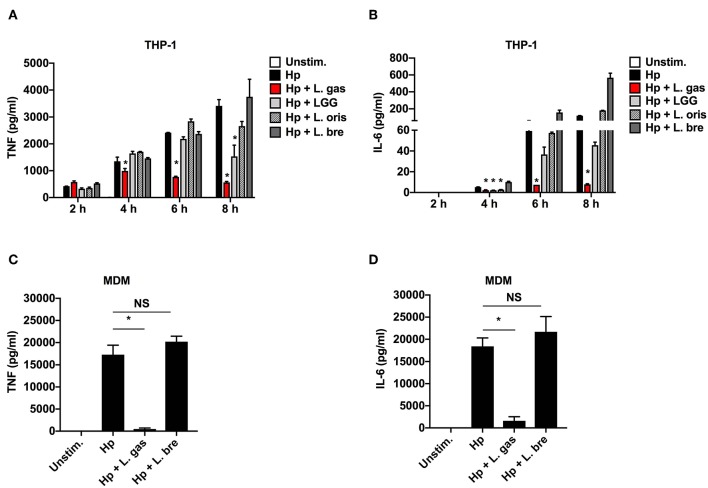
Certain strains of *Lactobacillus* inhibit the production of *H. pylori*-induced TNF and IL-6 in host macrophages. Human macrophages were infected with either *H. pylori* alone or in combination with lactobacilli. **(A)** TNF and **(B)** IL-6 were measured by ELISA in cell culture supernatants after 2, 4, 6, and 8 h of stimulation of THP-1-derived macrophages with Hp alone or in combination with *Lactobacillus* strains, L. gas, LGG, L. oris, or L. bre. *H. pylori* alone or in combination with L. gas or L. bre was incubated with MDMs for 8 h. **(C)** TNF and **(D)** IL-6 were determined by ELISA. The data presented are representative of results from at least three independent experiments with triplicate samples. Statistical analyses were performed using ANOVA (analysis of variance), followed by Bonferroni posttest. Error bars indicate standard deviation. ^*^P < 0.05 compared with *H. pylori* alone. Unstim., unstimulated.

To investigate the anti-inflammatory activity of lactobacilli, we quantified the levels of TNF and IL-6 produced upon stimulation of THP-1-derived macrophages with *H. pylori* alone or in the presence of different strains of *Lactobacillus*. *H. pylori* alone (Hp) induced the release of the proinflammatory cytokines TNF and IL-6 in a time-dependent manner (Figures 1A,B). However, when *H. pylori* was coincubated with L. gas, a significant reduction in the production of these cytokines was observed at all time points tested, apart from 2 h. With the exception of *Lactobacillus rhamnosus* GG (LGG), which suppressed TNF production at 8 h, there was no significant reduction in the secretion of TNF and IL-6 when cells were coincubated with *H. pylori* in combination with the other *Lactobacillus* species tested. The *Lactobacillus*-mediated inhibition of the proinflammatory cytokines was confirmed in human monocyte-derived macrophages (MDMs) using two representative strains. Consistent with the results from the THP-1-derived macrophages, the *H. pylori*-induced secretion of TNF and IL-6 was significantly inhibited only during coincubation with L. gas but not with *L. brevis* (L. bre) (Figures 1C,D). In summary, these results demonstrate that L. gas, but not L. bre, is able to inhibit the production of TNF and IL-6 in *H. pylori*-infected macrophages.

### TNF Gene Expression Is Differentially Regulated by *L. gasseri* in Macrophages

**Figure 2:**
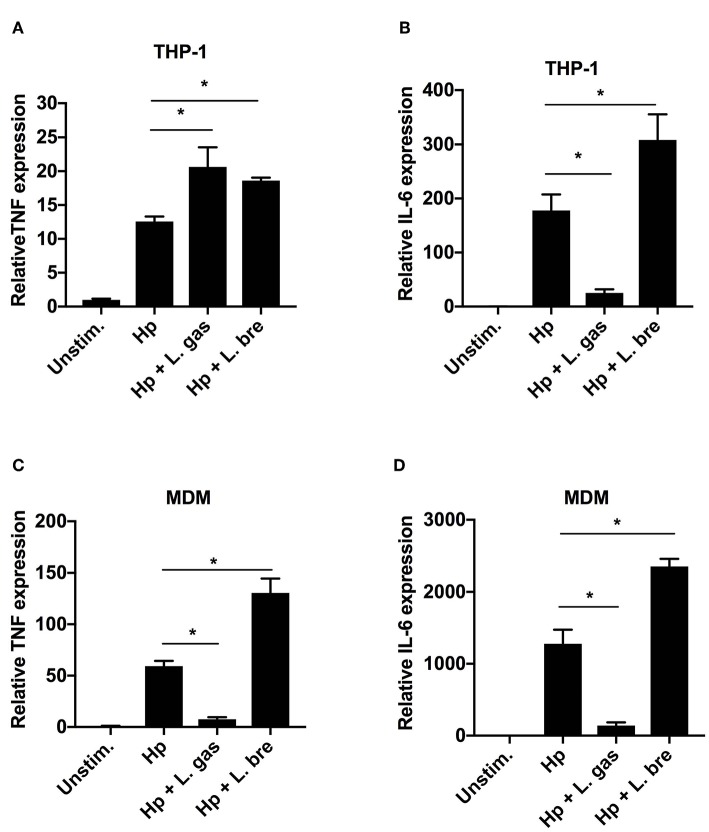
*Lactobacillus gasseri* regulates the gene expression of TNF in a cell type-specific manner. Human macrophages were infected with either Hp or in combination with L. gas or L. bre. RNA was extracted 8 h postinfection, and the expression of **(A)** TNF and **(B)** IL-6 in THP-1-derived macrophages and **(C)** TNF and **(D)** IL-6 in MDMs was analyzed by qPCR. Target mRNA levels were normalized to the reference gene *rpl37A* and are presented relative to the unstimulated control, which was set to 1. Data are the means and standard deviation of triplicate samples and are representative of three independent experiments. ^*^P < 0.05 using ANOVA followed by Bonferroni posttest.

To determine whether L. gas-mediated inhibition of TNF and IL-6 is regulated at the transcriptional level, we determined the expression levels of these cytokines by qPCR. As expected, Hp induced TNF and IL-6 gene expression in both THP-1-derived and MDMs (Figures 2A–D). When the cells were stimulated with a combination of Hp and L. gas, there was a significant increase in the transcription levels of TNF in THP-1-derived macrophages (Figure 2A), whereas TNF expression was significantly downregulated in the primary macrophages (Figure 2C). Furthermore, IL-6 expression was significantly downregulated by L. gas in both THP-1- and MDMs (Figures 2B,D). The non-inhibitory *Lactobacillus*, L. bre, did not suppress the expression of TNF and IL-6 during coincubation of cells with *H. pylori* (Figures 2A–D). These results suggest that lactobacilli affect gene expression in a species-specific manner, and that L. gas affects the gene expression of TNF in a cell-type-specific manner.

### A Heat-Sensitive Component of *L. gasseri* Exerts Anti-inflammatory Activity in a Contact and Dose-Dependent Manner

**Figure 3:**
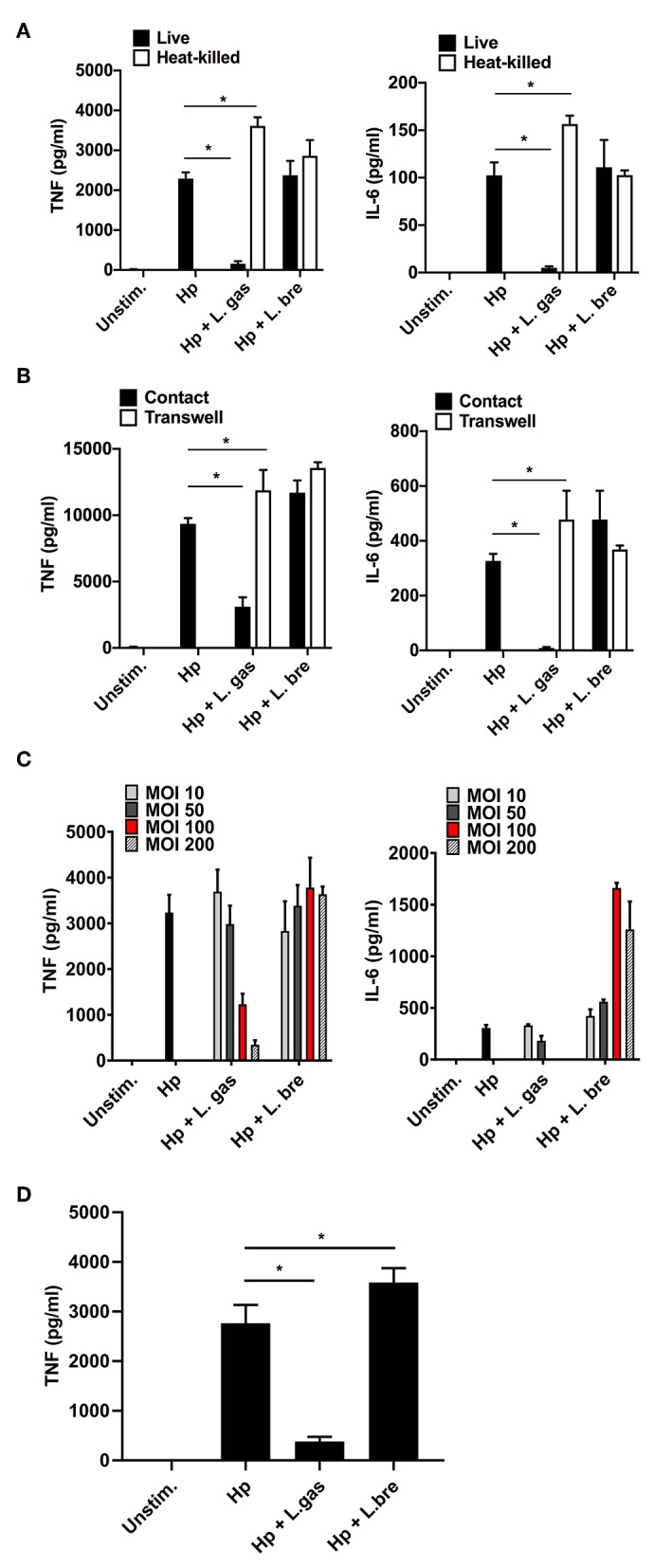
Suppression of *H. pylori*-induced TNF and IL-6 production by *L. gasseri* is contact and dose dependent and mediated by a heat-sensitive molecule. **(A–C)** THP-derived macrophages were incubated for 8 h with bacteria. **(A)** Effect of lactobacilli on the level of Hp-induced TNF (left panel) and IL-6 (right panel) when lactobacilli were live or heat-killed, **(B)** in contact or physically separated from the host cells and *H. pylori* using Millicell cell culture inserts. **(C)** The effect of different loads of lactobacilli upon Hp-induced TNF and IL-6 levels. MOI, multiplicity of infection. **(D)** Effect of lactobacilli on the level of Hp-induced TNF when THP-derived macrophages were pre-incubated with lactobacilli for 2 h before challenged with Hp for 8 h. ELISA was used to quantify TNF and IL-6. The presented data are representative of the means and standard deviation of at least three independent experiments performed in triplicate. Unstim., no bacteria added. ^*^P < 0.05.

To characterize the nature of the *Lactobacillus*-derived effector component(s) responsible for the immunomodulatory activity of L. gas, we treated lactobacilli with heat (95°C for 15 min) prior to stimulating THP-1-derived macrophages either with Hp or with *H. pylori* in the presence of live or heat-killed lactobacilli. In agreement with our previous results, live L. gas were able to reduce both TNF and IL-6 secretion in THP-1-derived macrophages. However, heat treatment completely abrogated the suppressive effect of L. gas on the production of these proinflammatory cytokines, suggesting the involvement of a heat-sensitive molecules (Figure 3A). Moreover, L. bre failed to reduce the production of TNF and IL-6, regardless of whether the lactobacilli were live or heat-killed. These results suggest that the inhibitory effect of L. gas might depend on bacterial viability.

We assessed whether the interplay between lactobacilli and host cells or lactobacilli and *H. pylori* is required to affect cytokine production. THP-1-derived macrophages were stimulated with (i) only *H. pylori*, (ii) simultaneously with *H. pylori* and lactobacilli, or (iii) with *H. pylori* in combination with lactobacilli, where the lactobacilli were physically separated from the host cells and *H. pylori* by Millicell cell culture inserts. Consistent with our previous data, L. bre did not suppress *H. pylori*-induced TNF and IL-6 production irrespective of bacterial contact (Figure 3B). However, L. gas that was in contact with host macrophages and *H. pylori* significantly decreased the production of TNF and IL-6. Separation of L. gas from the host cells and *H. pylori* abrogated the anti-inflammatory effect of L. gas (Figure 3B), indicating that an active interaction is essential for the inhibitory role played by L. gas.

Since we observed that direct contact was important for the inhibitory role of L. gas, we hypothesized that the reduction in TNF and IL-6 depends on the bacterial dose. To address this hypothesis, we incubated THP-1-derived macrophages with *H. pylori* or with *H. pylori* in the presence of lactobacilli at various multiplicities of infection (MOIs). The maximum inhibition of these cytokines was observed with the highest MOI of L. gas used for coincubation (Figure 3C). *H. pylori*-induced TNF and IL-6 secretion was not affected by L. bre, even though different bacterial MOIs were used.

We next examined whether pre-stimulation of macrophages with *Lactobacillus* strains prior to challenge with *H. pylori* could affect cytokine production. Here, THP-1-derived macrophages were first incubated with lactobacilli for 2 h and then challenged with *H. pylori*. The levels of TNF were significantly reduced when cells were prestimulated with L. gas prior to stimulation with *H. pylori*. However, there was no reduction in TNF levels in cells pre-incubated with L. bre (Figure 3D). This also matches to the cytokine inhibition pattern observed during simultaneous activation of host macrophages with *H. pylori* and lactobacilli.

### *Lactobacillus gasseri* Do Not Affect the Viability of *H. pylori* or Host Cells

**Figure 4:**
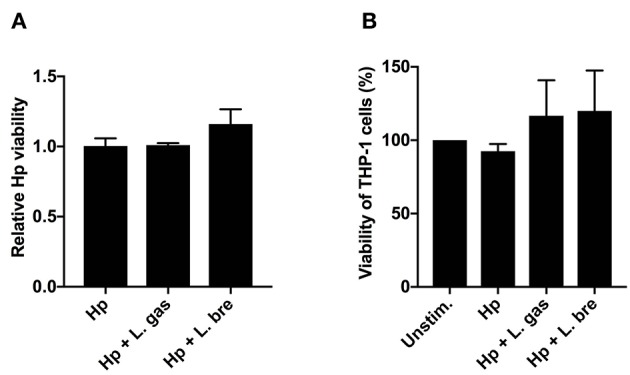
*Lactobacillus gasseri* do not affect the viability of *H. pylori* or host cells. THP-1-derived macrophages were infected with either Hp or in combination with L. gas or L. bre for 8 h. **(A)** The viability of *H. pylori* by viable count assay and **(B)** the viability of THP-1-derived macrophages by MTT assay was determined. Data are the means and standard deviation of triplicate samples and are representative of two independent experiments.

Lactic acid produced from various strains of *Lactobacillus* has been shown to exert a bactericidal activity against *H. pylori* (38–40). Hence, we evaluated the viability of *H. pylori* to assess whether the immunomodulatory activity of L. gas was the result of a lactic-acid-mediated bactericidal effect. The viability of *H. pylori*, however, was not affected by L. gas upon coincubation of host cells with the pathogen and lactobacilli, which excludes killing as a mechanism of cytokine inhibition (Figure 4A). To eliminate the possibility that changes in pH from lactic acid production could cause deleterious effects on host cells, we also analyzed the viability of THP-1-derived macrophages after infecting the cells with *H. pylori* or with *H. pylori* together with lactobacilli. Compared to the unstimulated cells, THP-1-derived macrophages were equally viable when incubated with bacteria (Figure 4B). Overall, these data demonstrate that L. gas does not affect the viability of *H. pylori* and host macrophages to block cytokine production.

### *Lactobacillus gasseri* Directly Affects Host Cells, and Its Anti-inflammatory Effect Is Not *H. pylori*-Specific

**Figure 5:**
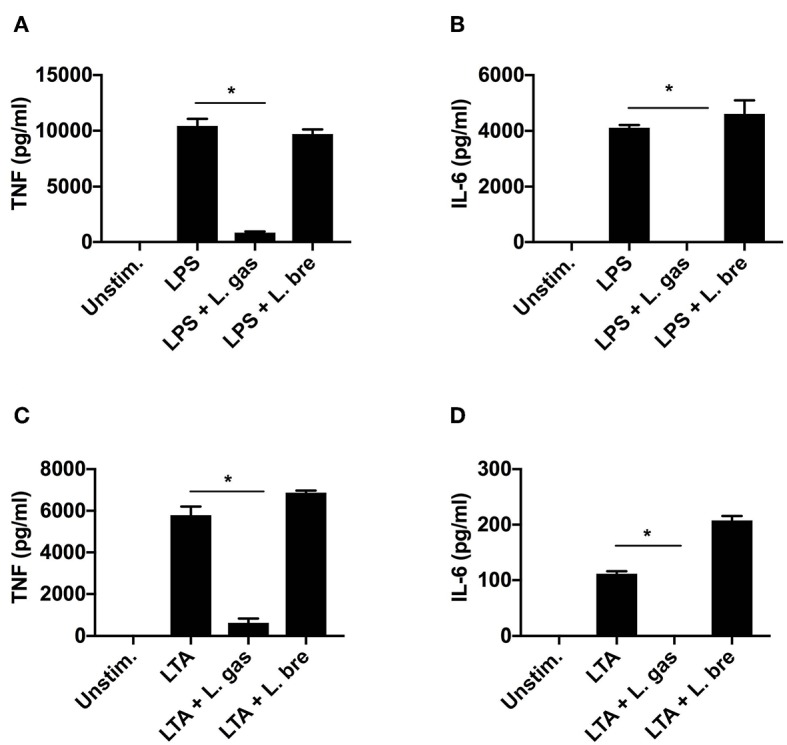
*Lactobacillus gasseri* blocks the induction of TNF and IL-6 in THP-1-derived macrophages stimulated either with LPS (1 μg/ml) or LTA (1 μg/ml). LPS or LTA alone or in combination with *Lactobacillus* strains was added to cells for 8 h. **(A)** TNF and **(B)** IL-6 production was determined by ELISA after coincubation of THP-1-derived macrophages with LPS alone or in combination with lactobacilli. **(C)** TNF and **(D)** IL-6 production was determined by ELISA after coincubation of THP-1-derived macrophages with LTA alone or together with lactobacilli. ^*^P < 0.05. Data are the means and standard deviation of triplicate samples and are representative of three independent experiments.

Since we have shown that direct contact between L. gas and *H. pylori* or between L. gas and host macrophages is necessary for the inhibition of TNF and IL-6 production by L. gas, we sought to determine whether L. gas affects *H. pylori* or host macrophages. Therefore, we treated THP-1-derived macrophages with known inducers of proinflammatory cytokines, LPS (1 μg/ml) or LTA (1 μg/ml) with or without *Lactobacillus* strains. Interestingly, when host cells were coincubated with LPS and L. gas, a significant reduction in TNF and IL-6 production was observed (Figures 5A,B). A similar inhibitory effect was observed during the coincubation of host macrophages with L. gas and LTA (Figures 5C,D). This significant inhibition of the secretion of TNF and IL-6 was not observed when host cells were treated with LPS or LTA in the presence of L. bre (Figures 5A–D). Together, these data show that the anti-inflammatory effect of L. gas is not *H. pylori*-specific and that L. gas affects host cells.

### *Lactobacillus gasseri* Does Not Affect Macrophage Polarization

**Figure 6:**
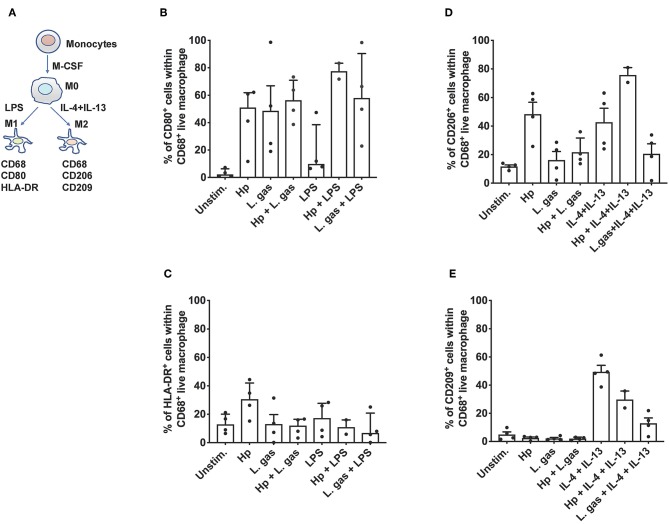
Differential effect of *H. pylori* and *L. gasseri* on macrophage polarization. **(A)** Schematic presentation of the polarization pathways of classically activated M1 and alternatively activated M2 macrophages. Monocytes isolated from the PBMCs of healthy donors were cultured with M-CSF containing medium for 6 days for differentiation into the M0 state. M1 and M2 macrophages were generated by treating M0 cells with LPS or with IL-4 and IL-13, respectively. M0 cells were also stimulated either with *H. pylori* or L. gas alone or in combination. Cells were then assessed with the following set of markers: **(B)** CD80, **(C)** HLA-DR, **(D)** CD206, and **(E)** CD209 by flow cytometry.

Next, we aimed to determine whether coincubation of cells with lactobacilli skews macrophages from the proinflammatory (M1) phenotype to the anti-inflammatory (M2) phenotype. To assess this question, we stimulated MDMs with either *H. pylori* or L. gas, alone or in combination, and evaluated M1 and M2 polarization markers by flow cytometry. The polarization protocol, with LPS and IL-4 plus IL-13 as M1 and M2 polarizing control stimuli, respectively, is shown in Figure 6A. Hp and L. gas were both found to be potent inducers of CD80 in human macrophages (Figure 6B), while *H. pylori* was found to be the strongest inducer of HLA-DR. Here, L. gas seemed to reduce the percentage of HLA-DR-positive cells, but the reduction was not significant (Figure 6C). Furthermore, we found that CD206 (M2 marker) was readily induced in the presence of Hp to a similar level obtained with the positive control IL-4 plus IL-13 (Figure 6D). Notably, the expression of CD206 was reduced upon coincubation of cells with L. gas, either in combination with Hp or IL-4 and IL-13 (Figure 6D). None of the bacteria induced CD209 expression but did reduce the percentage of CD209-positive cells in IL-4- and IL-13-polarized macrophages (Figure 6E). Taken together, these results suggest that the L. gas-mediated anti-inflammatory effect does not involve a general M1-M2 switch in human primary macrophages.

### *Lactobacillus gasseri* Affects the Production of TNF and IL-6 in *H. pylori*-Infected Macrophages by Inhibiting the Expression of ADAM17

**Figure 7:**
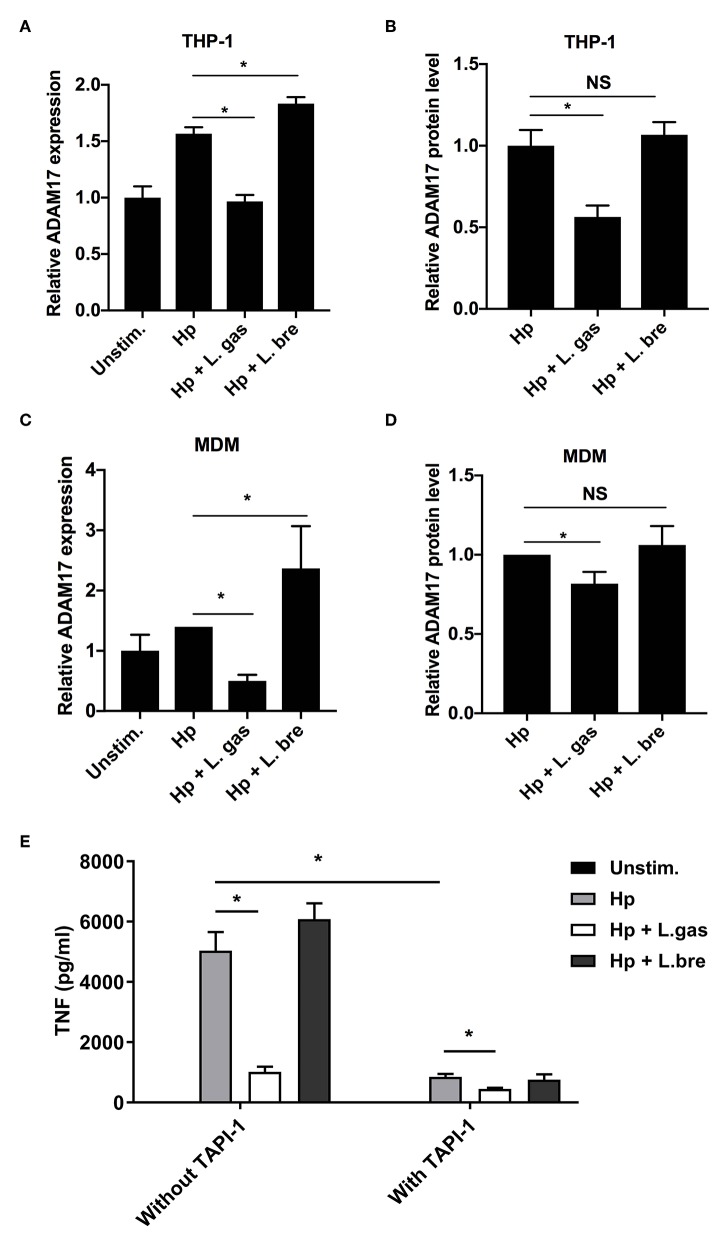
*Lactobacillus gasseri* prevents the production of TNF and IL-6 by inhibiting ADAM17 (a disintegrin and metalloproteinase 17). The gene expression and protein levels of ADAM17 were examined after incubation of THP-1-derived macrophages or MDMs with Hp or in combination with L. gas or L. bre. **(A)** mRNA levels of ADAM17 and **(B)** protein levels of ADAM17 in THP-1-derived macrophages were determined using qPCR or ELISA after 8 h of infection, respectively. **(C)** mRNA levels of ADAM17 and **(D)** protein levels of ADAM17 in MDMs were quantified using qPCR or ELISA at 8 h postinfection, respectively. **(E)** Protein levels of TNF in THP-1-derived macrophages were determined using ELISA after incubation of cells with or without TAPI-1 (50 μM). ^*^P < 0.05. Data are the means and standard deviation of triplicate samples and are representative of three independent experiments. NS, non-significant.

We investigated the expression of ADAM17 in infected cells at both the transcriptional and protein levels. Hp induced the expression of ADAM17 both at the mRNA and protein levels in THP-1-derived macrophages, while this induction was significantly reduced during infection with *H. pylori* in the presence of L. gas (Figure 7A,B). Coincubation of THP-1-derived macrophages with *H. pylori* and L. bre significantly upregulated ADAM17 expression at the transcriptional level but did not affect the protein level of the enzyme. A similar effect was observed in MDMs, where L. gas, but not L. bre, inhibited the *H. pylori*-induced expression of ADAM17 (Figures 7C,D). To determine whether suppression of ADAM17 expression is responsible for the observed L. gas effect on cytokine production, we inhibited ADAM17 in THP-1 macrophages using TAPI-1. TNF release induced by *H. pylori* was significantly reduced in macrophages treated with TAPI-1, confirming the importance of ADAM17 in TNF production. Further, L.gas reduced TNF to the same level as TAPI-1, suggesting that ADAM17 activity was blocked to a similar level by both systems. Adding both TAPI-1 and L.gas together slightly, but significantly, further reduced the TNF level, indicating a small additive effect (Figure 7E). In control experiments, TAPI-1 did not affect the protein level of IL-6 (Figure S1A) and did not change the mRNA level of TNF and IL-6 (Figure S1B). The inhibitory antibody D1(A12) to ADAM17 showed a similar inhibition as TAPI-1 (Figure S1C), supporting a role of ADAM17 in L. gas-mediated inhibition of *H. pylori* induced TNF. Further, addition of recombinant TNF to L. gas-inhibited *H. pylori* response indicated no change in IL-6 as determined by ELISA (Figure S1D). In summary, these data demonstrate a novel immunomodulatory mechanism, whereby L. gas blocks the production of the proinflammatory cytokines TNF and IL-6.

## Discussion

Chronic inflammation is one of the main factors that contribute to the development of various types of cancers. Infection with *H. pylori* induces long-lasting gastric inflammation and is the primary risk factor for the development of stomach cancer (41, 42). *Lactobacillus* species, on the other hand, have been reported to improve *H. pylori*-induced gastric inflammation (31, 43, 44). However, the mechanisms by which *Lactobacillus* strains achieve this role remain largely unknown. The objective of this work was to elucidate whether and how lactobacilli modulate the inflammatory response induced by *H. pylori* in human macrophages. In a previous study, we showed the ability of a human gastric isolate, *L. gasseri* Kx110A1 (L. gas), to prevent the attachment of *H. pylori* to host gastric epithelial cells and inhibit the colonization of the pathogen in an *in vivo* model (25). Here, we extend previous findings and show the *H. pylori*-independent anti-inflammatory properties of L. gas.

A strain-specific inhibition of pathogens by lactobacilli has been reported previously (45, 46). Similar to these reports, we found that only one out of four tested *Lactobacillus* strains was able to consistently reduce the secretion of TNF and IL-6 in *H. pylori*-infected macrophages, supporting the differential immunomodulatory role between *Lactobacillus* species. Interestingly, L. gas was isolated from human gastric biopsy and its adaptation to the niche might contribute to its ability to inhibit *H. pylori*-induced cytokines, however this is not the primary factor since the other gastric isolate, L. oris, did not affect cytokine production. In addition, L. gas was able to inhibit both TNF and IL-6 release when cells were stimulated with LPS or LTA, showing a general anti-inflammatory property. This inhibitory effect of L. gas was obtained via a direct interaction with the host macrophages.

Proinflammatory cytokines play a key role in exaggeration of inflammation and polymorphisms in cytokine genes are closely associated with increased risk of gastric cancer (47, 48). Dysregulation of the immune response by *H. pylori*, leading to abnormally increased levels of proinflammatory cytokines such as TNF and IL-6, has been reported to produce sustained inflammation and ultimately lead to gastric carcinogenesis (49, 50). Therefore, we examined the expression levels of these cytokines in *H. pylori*-infected macrophages in the presence or absence of lactobacilli. qPCR data revealed that L. gas blocked the gene expression of *H. pylori*-induced IL-6 in both THP-1-derived and MDMs, while *H. pylori*-induced TNF was differentially regulated in these cell types. This finding could indicate that the strain uses different mechanisms to suppress TNF in these two cell types. Although *H. pylori*-induced TNF was not reduced at the transcriptional level by L. gas in THP-1-derived macrophages, the inhibition of TNF at the protein level could be an indirect effect of the prevention of ADAM17 expression or the result of a posttranscriptional modification of the cytokine level. ADAM17 has been reported to be highly expressed in *H. pylori*-infected patients and in patients with gastric carcinomas (51). The fact that lactobacilli reduce the expression of *H. pylori*-induced ADAM17 is interesting and it opens for research surrounding the investigation of ADAM17 regulation and further understanding its mode of action. Moreover, it would be intriguing to study the involvement of various signaling pathways by the shed substrates. In this study, the level of *H. pylori*-induced TNF was significantly decreased when ADAM17 was inhibited, confirming its role in TNF production.

ADAM17 was first identified by its ability to cleave surface-bound pro-TNF to a soluble form (33). Currently, over 80 proteins have been identified as substrates for ADAM17, including the IL-6 receptor (IL-6R) (52). The cleavage and release of the IL-6R by ADAM17 initiates a process called IL-6 trans-signaling when the soluble receptor binds to its ligand IL-6 and initiates intracellular signaling. In classical IL-6 signaling, where IL-6 binds to the membrane-bound IL-6R, the cytokine is involved in homeostatic and anti-inflammatory processes. However, in IL-6 trans-signaling, the cytokine regulates proinflammatory reactions. Not all cells express IL-6R on their surface; in fact, IL-6R is primarily present on hepatocytes, some epithelial cells and a few types of leukocyte, limiting the number of cells that can respond to IL-6. However, through the process of IL-6 trans-signaling, cells that do not express IL-6R (nearly all cells in the body) can respond to the cytokine. This occurs when IL-6 binds and forms a complex with soluble IL-6R and thereafter binds to a protein called glycoprotein 130 (gp130) that is ubiquitously present on all cells (52–54). Inhibition of IL-6 trans-signaling has been shown to protect animal models from developing liver cancer and sepsis-associated mortality (55, 56). Since ADAM17 is responsible for releasing IL-6R and initiating IL-6 trans-signaling, it is intriguing that L. gas prevented the expression of *H. pylori*-induced ADAM17 in both THP-1-derived macrophages and MDMs. Furthermore, the inhibition of TNF via ADAM17 alleviates cytokine-mediated inflammation, reduces the risk of gastric cancer and may also prevent hypochlorhydria (4, 57). Because ADAM17 controls a wide array of genes, it would be interesting to study the anti-inflammatory role of L. gas in different disease conditions in the future.

Steric hindrance is one of the mechanisms by which lactobacilli prevent contact between pathogens and host cells (20, 58). Our results have shown that the anti-inflammatory effect of L. gas was dependent on lactobacilli*-*host cell contact, suggesting that an active interaction between lactobacilli and host macrophages is crucial for the observed effect. In addition, this finding indicates that the inhibitory effect of L. gas is not mediated by a molecule released into the environment but might be through an intact bacterial surface component(s). Together, these data indicate that lactobacilli could block *H. pylori* from binding to host cell receptors. We also demonstrated that heat-killed lactobacilli were not capable of reducing the production of TNF and IL-6 in macrophages, showing the involvement of a heat-sensitive molecules in the inhibitory action. Furthermore, we observed a dose-dependent reduction of proinflammatory cytokines by lactobacilli, where the highest MOI of lactobacilli used reduced these cytokines to the greatest extent, suggesting that the more lactobacilli that interact with host cells, the better the effect on cytokine production is. It is tempting to speculate that the L. gas effector molecule could possibly be a bacterial surface protein(s), but further investigation is needed to identify and characterize the effector molecule.

*Lactobacillus* strains inhibit the growth of various pathogens, including *H. pylori*, through the production of antimicrobial substances, such as bacteriocins and hydrogen peroxide. Moreover, changes in pH due to lactic acid secretion have previously been found to exhibit a bactericidal effect against *H. pylori* (20, 59, 60). We have shown that there was no reduction in the viability of *H. pylori* during coincubation with L. gas, confirming that the reduction in cytokine production was not a result of the killing of the pathogen.

Studies on human macrophage polarization have shown that macrophages exist as a mixed population of M1 and M2 macrophages instead of forming one distinct phenotype. In addition, macrophages can also switch between M1 and M2 phenotypes depending on the conditions of the local environmental milieu (61–64). Consistently, our flow cytometry results revealed that the expressions of M1 and M2 surface markers was differentially regulated by the stimuli used in the presence or absence of L. gas, but we did not observe one distinct phenotype.

In conclusion, our data provide novel insight into the immunomodulatory role of lactobacilli. In the future, it would be intriguing to study the anti-inflammatory role of L. gas in available animal models and compare its effect in acute and chronic *H. pylori* infection. Furthermore, identifying and characterizing the component of L. gas that is responsible for the inhibition of ADAM17 expression may allow the lactobacilli-derived component to be used as a potential treatment against various inflammatory diseases.

## Data Availability Statement

The datasets generated for this study are available on request to the corresponding author.

## Author Contributions

HG, KQ, TS, SP, HS, ES, and A-BJ planned experiments and wrote the manuscript. HG, KQ, and TS performed experiments. HG, KQ, TS, HS, ES, and A-BJ analyzed data.

### Conflict of Interest

The authors declare that the research was conducted in the absence of any commercial or financial relationships that could be construed as a potential conflict of interest.
