# Supplementary material for: Lactobacillus gasseri Suppresses the Production of Proinflammatory Cytokines in Helicobacter pylori-Infected Macrophages by Inhibiting the Expression of ADAM17
Source: Front Immunol. 2019 Oct 4;10:2326. doi: 10.3389/fimmu.2019.02326 (PMC6788455; doi:10.3389/fimmu.2019.02326)
Supplement: Supplementary file 1 [file Data_Sheet_1.PDF]

Supplementary Material

Lactobacillus gasseri **suppresses the production of proinflammatory cytokines in** ***Helicobacter pylori*****-infected macrophages by inhibiting the expression of ADAM17**

Hanna G. Gebremariam[1], Khaleda Rahman Qazi[1#], Tanvi Somiah[1#], Sushil Kumar Pathak[1,2], Hong Sjölinder[1,3], Eva Sverremark Ekström[1] and Ann-Beth Jonsson[1*]

[*] Corresponding author: Ann-Beth Jonsson, E-mail: ann-beth.jonsson@su.se

# These authors have contributed equally to this work

Supplementary figure 1

A

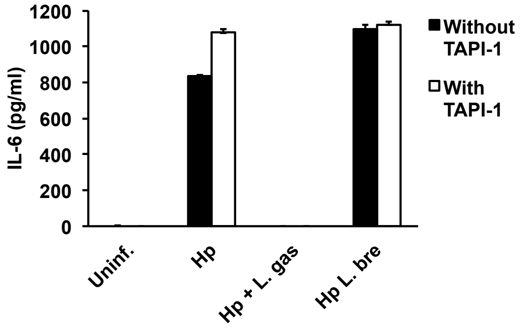

B

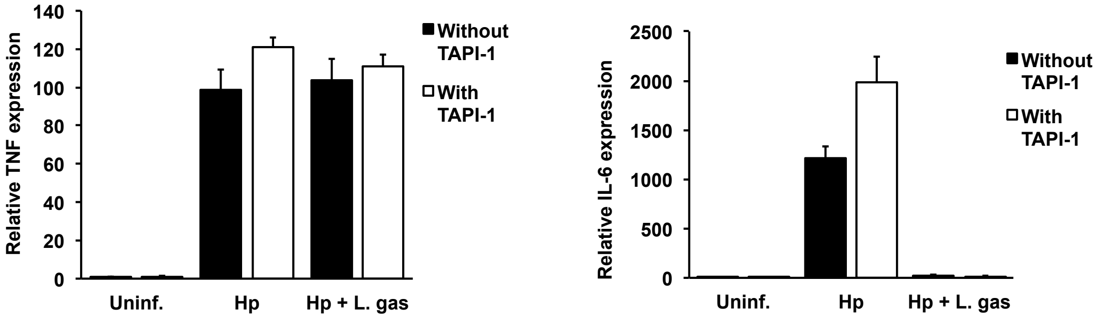

C

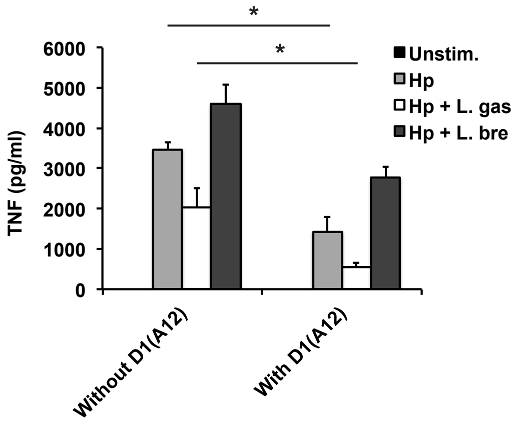

D

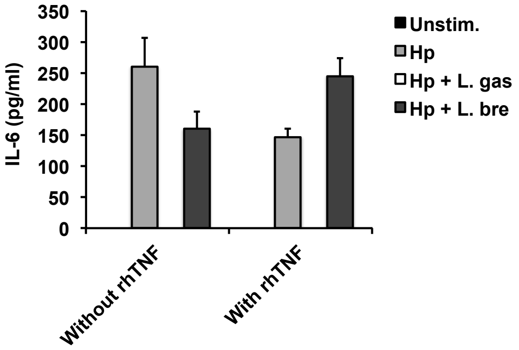

Figure S1. Control experiments supporting a role of ADAM17 in L. gas-mediated inhibition of Hp-induced TNF.

A) ELISA showing IL-6 production from THP-1-derived macrophages incubated with Hp or Hp+L.gas /L.bre with or without 50 μM TAPI-1.

B) mRNA levels of TNF and IL-6 in THP-1-derived macrophages incubated with Hp or in combination with L. gas with or without TAPI-1 for 8 h determined by qPCR.

C) Protein level of TNF in THP-1-derived macrophages incubated with Hp or Hp+L.gas with or without the ADAM17 inhibitory antibody D1(A12) (0.1 μM, Abcam) determined by ELISA.

D) Protein level of IL-6 in THP-1-derived macrophages incubated with Hp or Hp+L.gas with or without recombinant TNF (10 ng/ml, ImmunoTools) determined by ELISA. *, $P < 0.05$. Data are the means and standard deviation of duplicate samples in independent experiments. NS, nonsignificant.
